# A Dual Level Analysis with Evolutionary Computing and Swarm Models for Classification of Leukemia

**DOI:** 10.1155/2022/2052061

**Published:** 2022-05-26

**Authors:** Sunil Kumar Prabhakar, Semin Ryu, In cheol Jeong, Dong-Ok Won

**Affiliations:** Department of Artificial Intelligence Convergence, Hallym University, Chuncheon, 24252 Gangwon, Republic of Korea

## Abstract

One of the major reasons of mortality in human beings is cancer, and there is an absolute necessity for doctors to identify and treat a person suffering from it. Leukemia is a group of blood cancers that usually originates in the bone marrow and results in very high number of abnormal cells. For the diagnosis of cancer, microarray data serves as an important clinical application and serves as a great aid to the entire medical community. The dimensionality of the microarray data is too high, and so selection of suitable genes is quite an important step for the improvement of data classification. Therefore, for the prediction and diagnosis of cancer, there is an utmost necessity to select the most informative genes. In this work, Minimum Redundancy Maximum Relevance (MRMR), Signal to Noise Ratio (SNR), Multivariate Error Weight Uncorrelated Shrunken Centroid (EWUSC), and multivariate correlation-based feature selection (CFS) are chosen as initial feature selection techniques. Then, to select the most informative genes, five different kinds of evolutionary optimization techniques too are incorporated here such as African Buffalo Optimization (ABO), Artificial Bee Colony Optimization (ABCO), Cockroach Swarm Optimization (CSO), Imperialist Competitive Optimization (ICO), and Social Spider Optimization (SSO). Finally, the optimized values are fed through classification process and the best results are obtained when multivariate CFS with SSO is utilized and classified with Probabilistic Neural Network (PNN), and a high classification accuracy of 95.70% is obtained.

## 1. Introduction

One of the worst diseases which causes a lot of deaths in humans is cancer [1]. There are various types of cancer, and it causes the cells to divide in an uncontrollable manner, resulting in tumors, complete breakdown to the immune system, and impairments of vital organs [2]. Some kinds of cancer cause a rapid cell growth while others cause cells to grow at a slow rate. Some forms of cancer result in visual growths named tumors while others such as leukemia do not. One of the three different blood cancer forms is leukemia while lymphoma and myeloma are the other two forms of blood cancer [3]. An abnormal number of immature white blood cells is produced by leukemia which collapses the bone marrow and prevents the promotion of healthy important blood cells required for developing a balanced immune system [4]. The onset of acute leukemia is rapid and progresses very fastly, and therefore, urgent treatment has to be provided to them. Thus, leukemia belongs to a broad array of cancer disease and is commonly termed as hematological malignancies. There are two types here such as Acute Myeloid Leukemia (AML) and Acute Lymphoblastic Leukemia (ALL) [5].

*AML:* This kind of leukemia is the most prevalent type in older people but can affect younger people too. Due to the excess accumulation of immature hematopoietic cells in the blood and bone marrow, the malignancy occurs. Various genetic factors are responsible for such conditions.

*ALL:* This kind of disease is prevalent in children who are suffering from leukemia. When immature lymphoid cells excessively accumulate in the bone marrow and peripheral blood, this disease occurs.

Based on their morphological appearance, the categorization of the leukemia cells has been done traditionally. To identify the innate differences between tumor cells, there is an absolute necessity for highly skilled technological resources [6]. Such a process can be very expensive, highly time consuming, and tedious to handle. In a morphological manner, the cells can appear as similar, but each cell can respond quite differently to appropriate drugs and therapy [7]. Therefore, traditional techniques have huge limitations, and therefore, it leads to a necessity to identify other parameters so that cell categorization can be well framed [8]. High amount of useful information is provided by the gene expression data for subclassification studies. For managing gene expression data of thousands of genes simultaneously, microarrays have played quite an important role in it [9]. In the previous decade, microarray technology has been the most commonly used gene quantification method and is still in use due to the cheap and inexpensive nature of this technology [10]. Thus, using microarray techniques, the expression levels for tens of thousands of genes can be measured easily so that a functional relationship information is provided to the scientists between the physiological and cellular process of the biological organisms and genes [11]. As the microarray data is so huge to process owing to its large amount of noise and other disturbances, the curse of dimensionality problem is present and so gene selection is important so that the best genes are selected and provided for classification [12]. Some of the most important works done in leukemia microarray-based cancer classification are as follows:

For the diagnosis of chronic lymphocytic leukemia, Artificial Neural Network (ANN) was implemented by Aghamaleki et al. [13]. A novel prognostic classification of chronic lymphocytic leukemia derived from a multivariate survival analysis was done by Binet et al. [14]. ANN was utilized for recognizing and predicting leukemia by Afshar et al. [15]. Utilizing momentum back propagation and genetic algorithms as a feature selection technique, microarray-based leukemia classification was performed by Wisesty et al. [16]. The Leukemia diagnosis using transfer learning in Convolutional Neural Networks (CNNs) for classification was performed by Vogado et al. [17]. An effective Map Reduce-based KNN classifier was utilized for the analysis of microarray leukemia data by Kumar et al. [18]. An ensemble machine learning for leukemia cancer diagnosis based on microarray datasets was done by Alrefai [19]. A framework to detect and discriminate ALL and AML using microarray gene expression profiles utilizing supervised machine learning was done by Dwivedi [20]. To classify gene expression profiles of acute leukemia, various features and classifiers were explored by Cho [21]. An enhanced leukemia cancer classifier algorithm was done by Nasser et al. [22]. The application of Probabilistic Neural Network (PNN) to the class prediction of leukemia was done by Huang et al. [23]. Utilizing Partial Least Squares (PLS) method, the classification of acute leukemia based on DNA microarray gene expression was done by Nguyen et al. [24]. A SNR approach to discriminate AML with ALL was done by Goloub et al. [25]. Gene expression-based leukemia subclassification using committee neural network was found by Sewak et al. [26]. A leukemia multiclass assessment and classification from microarray and RNA-sequencing technologies integration at gene expression level was performed by Castiollo et al. [27].

Optimization algorithms have played a major role in gene selection procedure. An optimization-based tumor classification from microarray gene expression data was done by Dagliyan et al. [28], random cuckoo search for autism gene selection [29], and stellar mass black hole for engineering optimization etc [30]. Optimization models for cancer classification extracting gene interaction information from microarray expression was performed by Antonov et al. [31]. Other optimizations for cancer gene selection included a modified genetic algorithms with Levy flight [32], simplified swarm optimizations [33], chronological grasshopper optimization algorithms [34], Hybrid optimization algorithms [35], adaptive ant colony optimization [36], biogeography-based optimization [37], nondominated sorting GA [38], filter-based optimization [39], Particle Swarm Optimization (PSO) [40], Grey Wolf optimization [41], and hybrid of Grey Wolf and Crow search algorithm [42] have been reported in literature. In this work, the two-level feature selection employing statistical tests and then optimization techniques are done and then classified with suitable classifiers. The organization of the work is as follows. The experimental procedure is discussed in Section 2 along with the suitable gene/feature selection techniques.  Section 3 gives the details about the different optimization techniques, and Section 4 gives the classification techniques' details. The results and discussion are done in Section 5, and the paper is concluded in Section 6.

## 2. Materials and Methods

For the leukemia classification, a dataset was used which is publicly available online [25]. There are two types of leukemia, where 25 samples of acute myeloblastic leukemia (AML) and 47 samples of acute lymphoblastic leukemia (ALL) are found. The details of the dataset are tabulated in Table 1.

**Figure 1:**
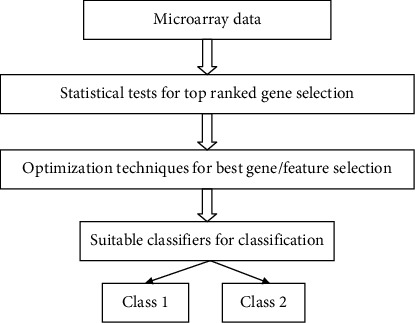
Illustration of the work.

The illustration of the work is shown in Figure 1.

### 2.1. Techniques to Select the Genes

The gene selection techniques utilized in this paper are as follows. The intention of this procedure is to shortlist the best 2000 genes from 7129 genes.

#### 2.1.1. Minimum Redundancy–Maximum Relevance (MRMR)

By means of minimizing redundancy, the features are selected with a maximum minimizing relevance [43]. To measure and assess the relevance for discrete datasets, a mutual information criterion is utilized by MRMR.

For a feature *Y*_*j*_, the F-test value is expressed by

(1) $$\begin{array}{c} F\left(Y_{j},S\right)=\frac{\left[\sum_{k}n_{k}\left(\mu_{jk}-\mu_{j}\right)\left(m-1\right)\right]}{\sigma^{2}}, \end{array}$$

where *S* = {*S*_*k*_} is the class set *k* = 1, 2, ⋯, *m*, *μ*_*j*_ represents the mean of *Y*_*j*_, *μ*_*jk*_ expresses the mean of *Y*_*j*_ for class *S*_*k*_, and *σ*^2^ = [*Σ*_*k*_(*n*_*k*_ − 1)*σ*_*k*_^2^]/(*n* − 1) represents the pooled variance for given size *n*_*k*_ and variance *σ*_*k*_^2^ of class *S*_*k*_. For feature subset *T*, the maximum relevance criterion is expressed by

(2) $$\begin{array}{c} \underset{t}{\max}\left[\frac{1}{\left|T\right|}\underset{j\in T}{\sum }F\left(Y_{j},S\right)\right]. \end{array}$$

The selection of the first method is done by this method, and utilizing the linear incremental search algorithm based on optimization function, the rest of the features are selected. However, for continuous variables, the two popular linear search schemes are MRMR-FDM and MRMR-FSQ schemes (F test distance multiplicative) and (F test similarity quotient).

For MRMR-FDM, the optimization condition is expressed as

(3) $$\begin{array}{c} \underset{j\in Y-T}{\max}\left[F\left(Y_{j},S\right).\frac{1}{T}\underset{q\in T}{\sum }d\left(Y_{j},Y_{q}\right)\right], \end{array}$$

where *d*(*Y*_*j*_, *Y*_*q*_) is the Euclidean distance between feature *Y*_*j*_ and *Y*_*q*_.

For MRMR- FSQ optimization,

(4) $$\begin{array}{c} \underset{j\in Y-T}{\max}\left[\frac{F\left(Y_{j},S\right)}{\left[1/\left|T\right|\sum_{q\in T}1/d\left(Y_{j},Y_{q}\right)\right]}\right]. \end{array}$$

#### 2.1.2. Signal to Noise Ratio

Pearson Correlation Coefficient (PCC) is quite an important measure utilized to find the gene significance. It is changed to specify the importance of SNR in using a gene as a predictor [44]. For a particular gene, to find the predictor strength, this predictor is utilized. For a gene ′*g*′, the calculation of SNR is done as

(5) $$\begin{array}{c} SNR\left(g\right)=\frac{\bar{y_{1}}-\bar{y_{2}}}{{sd}_{1}-{sd}_{2}}. \end{array}$$

The mean of the normal samples is expressed by $\bar{y_{1}}$, and the mean of the tumor sample is expressed by $\bar{y_{2}}$. *sd*_1_ and *sd*_2_ are the standard deviations of normal and tumor samples, respectively. The primary difference between the classes with respect to the standard deviation in between the classes is used by this value. Between the class distinction and the gene expression, a strong correlation is indicated if the values of *SNR*(*g*) are larger. If the values of *SNR*(*g*) are either positive or negative, then it corresponds to the gene being highly expressed in either class 1 or class 2. The genes which have a very large SNR value are quite informative, and so it is selected for cancer classification.

#### 2.1.3. Multivariate Error-Weighted Uncorrelated Shrunken Centroid (EWUSC)

Based on Shrunken Centroid (SC) and Uncorrelated Shrunken Centroid (USC), this technique was developed [45]. When the average gene expression for each gene in every class is divided by the standard deviation for that gene in the same class, then the Shrunken Centroid is found. Genes where expression is similar among the various samples of the same class, then higher weight is assigned to it. Using squared distance, to the label with the nearest average pattern, the assignment of new samples is done. From tracing the genes that are highly correlated in the set of genes found by SC, the redundant features are removed by USC approach. Both of these steps are used by EWUSC in addition to the error weights addition so that the redundant genes are removed, and the noisy genes are downgraded.

#### 2.1.4. Multivariate Correlation-Based Feature Selection (CFS)

When features are highly correlated with the class but uncorrelated with each other, then it forms a good feature subset [46]. By analyzing the predictive ability of every feature individually along with the degree of redundancy, the evaluation of a subset by CFS method is done. The main advantage of this technique is that a “heuristic merit” is provided for a feature subset instead of individual features. So, it implies that for a particular heuristic or function, the algorithm can decide on its progress by selecting the best options so that the output function is maximized.

## 3. Optimization Techniques

The shortlisted 2000 genes will undergo again a secondary feature selection methodology by means of utilization optimization techniques so that the best 50, 100, and 200 genes are finally considered and that is mentioned as a dual level analysis in this work. The feature selection is done using the five optimization algorithms as follows.

### 3.1. African Buffalo Optimization Algorithm

To get the best solution in the search space, ABO is utilized [47]. Within the herd population, the initialization of the buffaloes is done. Then, by updating their locations, the global optimum is searched for as they tend to follow the current best buffalo *bz*max in the herd. In the problem space, the buffaloes make sure it keeps track of its coordinates to achieve the best fitness value. The ideal location of the specific buffalo which is considered as the best with respect to the optimal solution is termed as *bq*max.*h*. Progressing towards *bq*max.*h* and *bz*max, the dynamic location of every buffalo is traced depending on where the importance is specified and kept at a particular location. The learning parameters has a great effect on the speed of each animal.

The ABO algorithm steps are explained as follows:

1. Initialization: the buffaloes are randomly placed to the different nodes of the solution space
2. Buffalo fitness value updation: the fitness value is updated as

(6) $$\begin{array}{c} f.h+1=f.h+lq1\left(bz\max-v.h\right)+lq2\left(bq\max.h-v.h\right) \end{array}$$

where *v*.*h* and *f*.*h* indicate the exploration and exploitation moves of the *h*^*th*^ buffalo (*h* = 1, 2, ⋯, *N*), *lq*1 and *lq*2 are learning factors, *bz*max is the best fitness of the herd, and *bq*max.*h* denotes the *h* best found location of the individual buffalo.

(3) The location of the buffalo *h* is updated utilizing the following formula as

(7) $$\begin{array}{c} v.h+1=\frac{\left(v.h+f.h\right)}{\pm 0.5} \end{array}$$

(4) If the updation of *bz*max is done, then proceed to step (5) or else go to (2) of this algorithm
(5) Check for the meeting of the stopping criteria. If met, go back to algorithm step (3) or else go to (6)
(6) The best solution is taken as the output

The updation equation (6) of the buffalo has 3 sections. The memory of the past location of the buffaloes is represented by *f*.*h*; a good memory ability is present for the buffalo which helps it to mention the places it has been before. This particular ability of the buffalo is important as it helps to search for best solutions by avoiding the areas that gave negative or poor results. As an alternative for the present local maximum location, a list of solutions is provided by the memory of each buffalo. The second part *lq*1(*bz*max − *v*.*h*) represents the cooperative nature of buffaloes and indicates the social nature of the buffaloes such as guarding each other, information sharing, and danger sensing. The third part *lq*2(*bq*max.*h* − *v*.*h*) mentions the intelligent abilities of the buffaloes. Therefore, the memory, socialization, and intelligent qualities of a buffalo are together represented in equation (6). Equation (7) helps the buffaloes in search of a better environment as the present environment has been fully explored and exploited or due to some unfavorable conditions.

The main highlights of the ABO algorithm are that to ensure a very fast convergence rate, and only a few parameters are used. In each iteration, the best buffalo *bz*max can be easily found out. To track the location and phase of the best buffalo (*bz*max), adequate exploration is ensured. By exploiting other buffalo's area too, a good exploration is achieved.

#### 3.1.1. Initialization and Updation of Speed and Location

In the solution space, by placing the *h*^*th*^ buffalo randomly, initialization phase is done. For the algorithm to converge in a smaller number of iterations, the previous knowledge of the problem can be helpful. Based on the previous maximum location (*bq*max) and source data gathered from the exploits of the other neighboring buffaloes, the updation of the location of every buffalo is done in each iteration. With such a modelling, the algorithm can track the buffalo movement to achieve an optimal solution.

### 3.2. Artificial Bee Colony Algorithm

In a multidimensional space, based on the bee's foraging activity for nectar, this global cum local search-based optimization procedure was utilized and the steps are explained in Algorithm 1 [48]. In this entire variable space, the food sources are spanning throughout, and in this variable space, the food source is assumed as the point in the variable space. For that particular point in the variable space, the objective function is maximized by this ABC method similar to the location tracing of the food source by the bee which has the highest nectar content. The objective function *f*(*y*) should find the optimal solution in this ABC optimization problem where in an artificial multidimensional space, and the artificial bees will wander to trace the highest producing nectar source. The search task is achieved by utilizing the basic concept of food foraging procedure by the bee colony and is simulated in an artificial computer surrounding. In the entire variable space, a random population of initial food sources is denoted as *y*^(*m*)^(*m* = 1, 2, ⋯, *N*), where *N* indicates the colony size is expressed as

(8) $$\begin{array}{c} y_{i}^{\left(m\right)}=y_{i}^{\left(H\right)}+v_{i}\left(y_{i}^{\left(V\right)}-y_{i}^{\left(H\right)}\right), \end{array}$$

where *v*_*i*_ is a random number in the range of [0,1]. Three different types of tasks are assured where each does a different task. A food source from their respective memories is considered by the employed bees and then seek a new food source *w*_*f*_ in its neighbourhood. For this purpose, any neighbourhood operator can be utilized. A food source which is uniformly distributed within ±*z* of the present memory location *y*_*n*_ is utilized as

(9) $$\begin{array}{c} w_{f,i}=y_{n,i}+\phi_{i}\left(y_{n,i}-y_{i}^{\left(m\right)}\right), \end{array}$$

where the randomly selected food source is expressed as *y*^(*m*)^, and *ϕ*_*i*_ is a random number in [−*z*_*i*_, *z*_*i*_]. The food source *w*_*f*_ which is newly created is then compared with *y*_*n*_. and the food source which is better is placed in the memory of the employed bee. Here in our experiment, the total number of employed bees is set as 60% of the total food sources (*S*). The food source information stored in their memories is shared by the employed bees with the onlooker bees who is present in the bee hive observing the foraging act of the employed bees. The food source location *w*_*f*_ traced by an employed bee is chosen by the onlooker bee in a probabilistic manner proportion to the total nectar content in the food source *w*_*f*_. The probability of choosing the food source is higher if the nectar content is high. Modification of a selected food source to trace *w*_0_ in its neighbourhood is done by using a similar methodology with the selected *w*_*f*_ as shown in equation (9). The memory of the onlooker bee selects and keep only the better of the two food sources. The number of onlooker bees is generally set as half of the food sources. Finally, the scout bees are the third kind of bees which chooses a food source location randomly utilizing equation (8) and act like global overseers. Though a predefined number of trials, if the memory location cannot be improved by the employed bees, then it booms as a mount bee. Once it becomes a scout bee, then in the variable space, the memory located is reinitialized randomly. The number of scout bees is assumed to be 1 in our experiment, and the algorithm runs for a maximum number of *G* generations. Only with an employed or an onlooker bee alone, each food source is associated, so that a single food source is associated in each of them. It is therefore used in other types of optimization too such as combinatorial optimization, multiobjective optimization, and to solve integer programming.

### 3.3. Cockroach Swarm Optimization Algorithm

Inspired by the nature of the cockroaches searching for food such as progressing in swarms, escape mechanisms, or scattering mechanism from light, CSO was developed [49]. The collective cockroach behaviour is modelled by a set of rules in the CSO algorithm. The focus of this algorithm is to create a set of feasible solutions in its initial step. In the search space, the random generation of the initial solutions are done. For solving various optimization problems, the CSO algorithm includes 3 procedures such as (i) chase swarming, (ii) dispersing, and (iii) ruthless behaviour.

#### 3.3.1. Chase-Swarming Phase

In this phase, the local best solutions *S*_*i*_ are carried by the strongest cockroaches and then together it forms a small swarm. After the swarm formation, it is progressed towards the global optimum *S*_*o*_. In this procedure, within the range of its visibility, each individual *A*_*i*_ progresses towards its local optimum. During the movement of the cockroaches in small groups, a particular approach can become the strongest by means of finding a better solution. Within its own visibility scope, a lonely cockroach has its local optimum and it progresses towards the global best solution.

#### 3.3.2. Dispersion of Individual Phase

To preserve the diversity of cockroaches, it is performed from time to time. In this phase, a random step is taken by the cockroach in the search space.

#### 3.3.3. Ruthless Behaviour Phase

Here, the currently best individuals replace the random individual. If the food availability is inadequate, then creating the weaker cockroaches becomes the procedure and so it is termed as ruthless behaviour.

The steps are as follows:

(Step 1) A population of ′*q*′ individuals is generated, and the algorithm parameters are initialized (step, D-space dimension, visual scope, and stopping criterion)
(Step 2) Within the visual scope of the *j*^*th*^ individual, *S*_*j*_ and *S*_*o*_ is searched for
(Step 3) Chase swarming behaviour is implemented, and finally *S*_*o*_ is updated at the end. If a cockroach *A*_*j*_ is local optimum, then it progresses to *S*_*o*_ based on *A*_*j*_ = *A*_*j*_ + *step*.rand.(*S*_*o*_ − *A*_*j*_), where rand is a random number within [0,1]. Or else the cockroach *A*_*j*_ progresses to *S*_*j*_ through the formula represented as

(10) $$\begin{array}{c} A_{j}=A_{j}+step.\mathrm{rand}.\left(S_{j}-A_{j}\right) \end{array}$$

and it is present within its own visibility range

(Step 4) Dispersing procedure is implemented, and *S*_*o*_ is updated
(Step 5) Ruthless procedure is implemented (*A*_*y*_ = *S*_*o*_) or (*A*_*y*_ = 0), where *y* = 1, ⋯, *q*
(Step 6) Until a termination criterion is satisfied, the steps 2-5 are repeated and then output the final results. The stopping criteria includes the computation time, obtaining a minimum solution error and maximum number of iterations etc

### 3.4. Imperialist Competitive Algorithm

One of the famously used population-based metaheuristic is ICA. In a population, each individual represents a country, and in the initialization process, some best countries are selected as imperialists [50]. The imperialist and colonies help to build the initial empire, and the generation of the new solutions is done by the colony assimilation and revolution, competition among the imperialists, and the exchange of imperialists.

The procedure is as follows:

1. Initialization: An initial population *P* is generated
2. Initial empire construction: The cost *t*_*i*_ is computed for every individual; for all the solutions, the sorting of *t*_*i*_ in descending order is done. The selection of *M*_*im*_ best solutions from *P* as imperialists is done. The remaining countries *M*_*col*_ is assigned to the imperialists
3. The assimilation of colonies is executed for every empire, and then the revolution of some colonies is performed. If possible, position of colony and imperialist is exchanged
4. The imperialist competition is achieved
5. Without any countries, the empire is eliminated
6. If the meeting of termination criteria is not done, then it goes back to step (3)
7. If the termination criteria are done, then the search is stopped

Based on the objective function, the calculation of the cost of a country is done. The cost is less if a solution is better. *M*_*im*_ best solutions with the least cost are considered as imperialists. The colonies are formed by the rest of the countries. There are totally *M*_*col*_ colonies represented as *M*_*col*_ = *M* − *M*_*im*_.

By the assignment of colonies to imperialists, the formation of initial empires is done based on the imperialist power and is considered as

(11) $$\begin{array}{c} P_{q}=\left|\frac{Z_{q}}{\sum_{w=1}^{M_{im}}Z_{w}}\right|, \end{array}$$

where *P*_*q*_ denotes the power of imperialist *q* and *Z*_*w*_ = max_*q*_{*t*_*q*_} − *t*_*w*_ denotes the normalized cost, and here, *t*_*q*_ specifies the imperialist cost of *q*. The calculation of the number of initial colonies managed by imperialist *q* is expressed as *round*{*P*_*q*_ × *M*_*col*_}, where round is the nearest integer of a fractional number and is expressed by the function round.

The total number of colonies of imperialist *q* is expressed by *S*_*q*_. A colony in each empire progresses *ε* along the ′*d*′ direction towards the imperialist in the process of assimilation. *ε* is the moving distance and is a random number represented by random distribution in the interval [0, *c* × *d*], where *c* ∈ (1, 2) and the distance among imperialist and colony is expressed by *d*. The colony progresses towards the direction of the imperialist if *c* > 1. However, the colonies cannot be absorbed by the imperialist in direct movement thereby a deviation from the direct line prevails. The representation of deviation is done by *θ* which follows uniform distribution in [−*φ*, *φ*], where *φ* is just an arbitrary parameter. Change in position of some colonies causes revolution, and it is because of unexpected changes in the characteristics. For instance, the change in characteristic would lead to the change in position, and it can be influence by changing the language or religion of a particular colony. Similar to the process of mutation in competitive algorithm, the revolution in ICA is carried out so that exploration is increased and the early convergence to local optima is prevented. Once the assimilation and revolution is done in an empire, the comparison of the cost of each colony with that of the imperialist is done. Therefore, if the colony has a very less cost in comparison to the imperialist, then the swapping of colony can be done. Depending on the total empire power, the determination of imperialist competition is done. Assume *AT*_*q*_ is the total cost of the empire *q*, therefore for each empire *q*, *AT*_*q*_ is initially calculated as

(12) $$\begin{array}{c} {AT}_{q}=t_{q}+\zeta \times \text{mean}\left\{\text{cost}\left(\text{colors of empire}\right)\right\}, \end{array}$$

where *ζ* represents a positive number between 0 and 1, and it is close to 0. For the empire *q*, the normalized cut cost and the power is computed as

(13) $$\begin{array}{c} \text{Normalized}{AT}_{q}=\underset{s}{\max}\left\{{AT}_{s}\right\}-{AT}_{q}, \end{array}$$

(14) $$\begin{array}{c} {EP}_{q}=\left|\frac{{MAT}_{q}}{\sum_{w=1}^{M_{im}}{MAT}_{w}}\right|, \end{array}$$

After a vector [*EP*_1_ − *c*_1_, *EP*_2_ − *c*_2_, ⋯, *EP*_*M*_*im*__ − *c*_*M*_*im*__] is defined, the assignment of the weakest colony from the weakest expires to the empire having largest index is done where *c*_*i*_ represents the random number with uniform distribution in the range of [0,1].

### 3.5. Social Spider Optimization Algorithm

One of the recent meta heuristic algorithm which attracted a good attention is SSO [51]. In this algorithm, the search space is assumed as a communal spider web. For each population, the candidate solutions represent a spider. A weight is received from each spider based on its fitness value. The simulation of the various cooperation behaviour in the colony is approached by two different search sets of evolutionary operators. To solve a nonlinear global optimum problem, the algorithm is designed with box constraint as follows:

Minimize: *f*(*y*), *y* = (*y*^1^, *y*^2^, ⋯, *y*^*d*^) ∈ *ℜ*^*d*^

Subject to *y* ∈ *Y*

where *f* : *ℜ*^*d*^⟶*ℜ* is a nonlinear function, and *Y* = {*y* ∈ *ℜ*^*d*^|*l*_*j*_ ≤ *y* ≤ *u*_*j*_, *j* = 1, . ⋯ , *d*} is a feasible space reduced by limiting the lower (*l*_*j*_) and upper (*u*_*j*_) limits. To solve this optimization problem, population *A* of *N* candidate solutions is utilized by SSO. A spider position is represented by each solution whereas the search space *Y* is represented by the general web. In this methodology, the population *A* is divided into two search agents. (*M*_*a*_) represent male and (*F*_*a*_) represent female. The real spider colony is aimed to be simulated and therefore the number of females (represented as *N*_*f*_) is selected randomly in the range of 60-70% of the entire population *A*, where the rest is considered as the male individuals (*N*_*m*_ = *A* − *N*_*f*_). Under this constraint, a set of female individuals is formed by the group *F*_*a*_ as (*F*_*a*_ = *f*_*a*1_, *f*_*a*2_, ⋯, *f*_*a*_*Nf*__), and the male individuals (*M*_*a*_ = *m*_*a*1_, *m*_*a*2_, ⋯, *m*_*a*_*Nm*__). Each spider ′*c*′ has a weight *w*_*c*_ based on solution fitness and it is calculated as

(15) $$\begin{array}{c} w_{c}=\frac{{fit}_{c}-worst}{best-worst}, \end{array}$$

where *fit*_*c*_ represents the fitness value of the *c*^*th*^ spider solution, *c* ∈ 1, ⋯, *N*, *best* indicates best fitness value, and *worst* indicate worst fitness value of the whole population *A*. The main mechanism of SSO is the information exchange in the optimization process. Only through the vibration present in the website it can be simulated. The modelling of a vibration received from a spider *b* to spider *c* is expressed as follows

(16) $$\begin{array}{c} V_{c,b}=w_{b}e^{d_{c,b}^{2}}, \end{array}$$

where the weight of the *b*^*th*^ spider is *w*_*b*_, and the distance between the 2 spiders is ′*d*′. Three types of vibrations can be perceived by each spider ′*c*′ and *v*_*c*,*n*_, *v*_*c*,*h*_ and *v*_*c*,*f*_.*v*_*c*,*n*_. The vibration produced by the nearest spider ′*n*′ with a very high weight is expressed by *v*_*c*,*n*_. *v*_*c*,*f*_ is produced by the closest female spider, and their vibrations applied only if *c* is a male spider. In the population *A*, the best spider is produced by *v*_*c*,*h*_. At an initial stage *s* = 0, a population *N* of the total spider is operated to assess the total number of iterations (*s* = *iterations*). Various sets of evolutionary operators are assigned to each individual based on its gender.

In the context of female spiders, the novel position *fa*_*c*_^*s*+1^ is obtained by the modification of the current position of spider *fa*_*c*_^*s*^. A probability factor *P* is used to randomly control the modification, and the movement is produced with respect to other spiders and throughout the search space, and the transmission of vibrations is done as

(17) $$\begin{array}{c} {fa}_{c}^{s+1}=\left\{\begin{array}{c} {fa}_{c}^{s}+\alpha .V_{c,n}.\left(a_{n}-{fa}_{c}^{s}\right)+\beta .V_{c,n}.\left(a_{h}-{fa}_{c}^{s}\right)+\delta .\left(\mathrm{rand}-\frac{1}{2}\right),\\ {fa}_{c}^{s}-\alpha .V_{c,n}.\left(a_{n}-{fa}_{c}^{s}\right)-\beta .V_{c,h}.\left(a_{h}-{fa}_{c}^{s}\right)+\delta .\left(\mathrm{rand}-\frac{1}{2}\right), \end{array}\right. \end{array}$$

with probability *P* and 1 − *P*, where *α*, *β*, *δ*, rand are random numbers between the range [0,1] and *s* denotes the iteration number. *a*_*n*_ and *a*_*h*_ are the nearest spider and best spider, respectively. The classification of male spiders in 2 types is done as dominant (*U*) and nondominant (*W*). Only between the dominant male *m*_*u*_ and female individuals, the mating is carried out with a specific range *r*, and so a new individual *a*_*new*_ is defined by the weight of each spider. The new individual *a*_*new*_ can be influenced easily by the heavier element which has more probability. Once the generation of the new spider is done, it is then compared to the rest of the population. If a new spider has a good fitness value than the worst spider member, then the worst spider is replaced by *a*_*new*_, or else discarding *a*_*new*_ is done.

## 4. Classification Procedures

The optimized values are then classified with the following classifiers.

### 4.1. NBC

The main assumption of NB classifier is that each characteristic is pretty independent to the rest of the characteristics [52]. Therefore, the optimized genes contribute in an independent manner to the probability of being a part of a specific class. For estimating the essential parameters for classification, a smaller number of training samples is required by these types of classifiers. For supervised learning problem, it is a fast and efficient classifier.

### 4.2. SVM

The main intention of SVM lies in the hyperplane selection that is equidistant from every class so that for the separation of the classes, a maximum margin is achieved [53]. The training support vector samples are the ones which fall into the frontier when the hyperplane is defined. The classifier greatly tolerates the classification errors which is controlled by the hyperparameters so that generalization capability of the model is controlled. Depending on the side of hyperplane to which the sample belongs, the classification of a new sample will be done for a biclass classification. This method usually changes for a multiclass classification because SVM builds (*N* − 1)∗*N*/2 classifiers where the number of classes is denoted by *N*. Then, a voting system is also established among them mentioning the most voted class for the new samples.

### 4.3. RF

A forest of classification trees is built by the RF algorithm as it grows many single classification vectors (trees) [54]. A vector is assigned as an input to be classified in this classification model for each tree of the forest. Once the classification is done by that individual tree, the class having the largest number of votes over all the trees is decided by the standard voting system among the trees.

### 4.4. PNN

This classifier is an implementation of a statistical algorithm called kernel discriminate analysis. The operations are usually organized into a multilayered feed forward network [55]. Only one epoch of training is needed in PNN. The main drawback of using this is that for storing the training samples, it assumes a lot of memory and so the recall process computation slows down gradually.

## 5. Results and Discussion

It is classified with a 10-fold cross validation method, and the performance of it is shown in tables below. The mathematical formulae for computing the Performance Index (PI), Sensitivity, Specificity, and Accuracy are mentioned in literature, and using the same, the values are computed and exhibited. PC is Perfect Classification, MC is Missed Classification, and FA is False Alarm in the expressions below.

The sensitivity is expressed as

(18) $$\begin{array}{c} \text{Sensitivity}=\frac{\text{PC}}{\text{PC}+\text{FA}}\times 100 \end{array}$$

Specificity is expressed as

(19) $$\begin{array}{c} \text{Specificity}=\frac{\text{PC}}{\text{PC}+\text{MC}}\times 100 \end{array}$$

Accuracy is expressed as

(20) $$\begin{array}{c} \text{Accuracy}=\frac{\text{Sensitivity}+\text{Specificity}}{2} \end{array}$$

Performance Index (PI) is expressed as

(21) $$\begin{array}{c} \text{PI}=\left(\frac{\text{PC}‐\text{MC}‐\text{FA}}{\text{PC}}\right)\times 100 \end{array}$$

Table 2 shows the average performance analysis of classifiers in terms of classification accuracies with ABO for different gene selection techniques using 50–200 selected genes. As depicted in Table 2, the PNN classifier with 50 genes at SNR features and PNN classifier with 200 genes selected in the multivariate EWUSC attained higher accuracy of 92.97%. In the case of SVM classifier with 100 genes for the multivariate, CFS reached a low accuracy value of 75.96%. This low accuracy is due to the high false alarm rate in the SVM classifier.

Table 3 demonstrates the average performance analysis of classifiers in terms of classification accuracies with ABCO for different gene selection techniques using 50–200 selected genes. As shown in Table 3, the PNN classifier with 200 genes at SNR feature exhibits higher accuracy of 91.47%. The SVM classifier with 200 genes for the multivariate EWUSC feature is ebbed at the low accuracy of 75.7581%.

Table 4 reveals the average performance analysis of classifiers in terms of classification accuracies with CSO for different gene selection techniques using 50–200 selected genes. As identified in Table 4, the NBC classifier with 50 genes at SNR feature demonstrates the higher accuracy of 92.19%. The PNN classifier with 50 genes for the multivariate EWUSC feature is achieved at the low accuracy of 75.75%.

Table 5 exposes the average performance analysis of classifiers in terms of classification accuracies with ICO for different gene selection techniques using 50–200 selected genes. The Table 5 reports that RF classifier with 50 genes at multivariate CFS attained the higher accuracy of 92.45%. The PNN classifier with 50 genes for the multivariate EWUSC feature is achieved at the low accuracy of 75.625%.

Table 6 expresses the average performance analysis of classifiers in terms of classification accuracies with SSO for different gene selection techniques using 50–200 selected genes. Table 6 exposes that PNN classifier with 200 genes at multivariate CFS attained the highest accuracy of 95.705%. The RF Classifier with 50 genes for the multivariate CFS feature achieved the lower accuracy of 75.875%.

**Figure 2:**
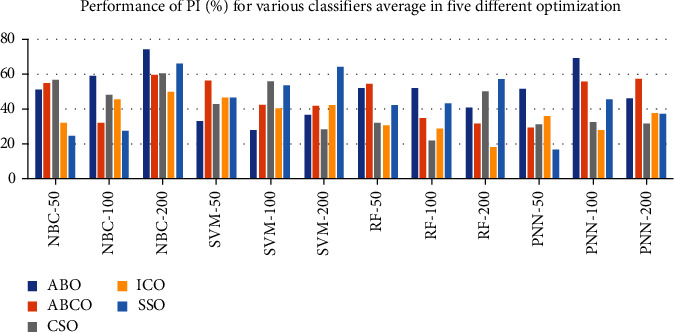
Performance of Performance Index for various classifier average in five different optimizations.

Figure 2 shows the performance of Performance Index (PI) parameter for four classifiers averaged in five different optimization methods. As exhibited in Figure 2, the NBC classifier with 50 gene selection at CSO optimization attained higher PI of 56.94%. As in the case of NBC classifier with 100 genes for ABO algorithm, the higher PI is reached at 59.03%. NBC classifier with 200 genes selection for ABO algorithm peaked with the highest PI of 74.33%. For SVM classifier with 50 gene selection ABCO algorithm edged at high PI of 56.33% and SVM with 100 genes depicted the PI of 55.93% for the CSO algorithm. In SVM classifier with 200 genes selection case, SSO algorithm reports high PI of 63.64%. RF classifiers with 50 genes selection procedure attained high PI of 54.68% at ABCO algorithms. As in the case of RF classifiers with 100 gene selection method, ABO algorithm provides higher PI of 51.56%. In the RF classifier with 200 genes selection method, SSO algorithm exhibits high PI value of 57.41%. In PNN classifier with 50 gene selection procedure, ABO algorithm arrived at high PI of 51.68%. For the PNN classifier with 100 genes selection method also, ABO specialized at higher PI of 69.32%. For PNN classifier with 200 genes selection cases good PI of 57.32% is reached for ABCO algorithm. Due to the averaging effect across the four features, the classifier reveals better and smooth PI values. ISO algorithms demonstrate the smoothening effect across the classifiers.

## 6. Conclusion and Future Work

For the diagnosis, analysis, and treatment of cancer, microarray-based classification of this disease is very useful. To determine the most informative genes that can cause cancer, a great impact and utility was provided by the microarray technique in recent years. The curse of dimensionality problem is a huge drawback in microarray data analysis which destabilizes the computational instability and prevents the usefulness of a certain information from a dataset. Thus, in analyzing the cancer microarray datasets, an imperative task lies in the selection and extraction of relevant features so that effective classification is achieved. In this work, four types of initial feature selection techniques were performed and then it was further optimized with five optimization techniques before proceeding into classification. The best results are obtained when multivariate CFS feature selection with SSO is utilized and classified with Probabilistic Neural Network (PNN), and a high classification accuracy of 95.70% is obtained. Future work is to analyze with a plethora of other optimization and machine learning techniques for a better analysis of microarray-based leukemia classification.

## Figures and Tables

**Algorithm 1:**
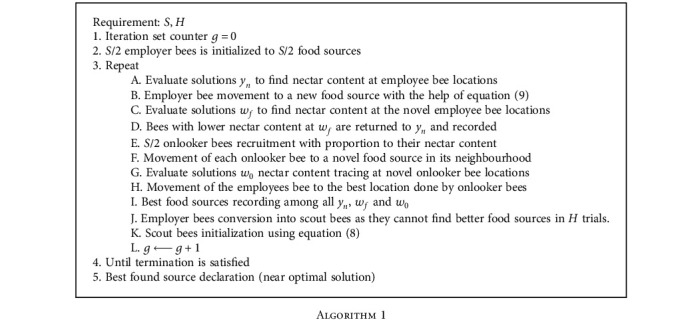


**Table 1:** Dataset details.

Dataset	Number of genes	Class 1 (ALL)	Class 2 (AML)	Total samples
Leukemia: AML-ALL	7129	47	25	72

**Table 2:** Performance analysis of classifiers in terms of classification accuracies with ABO for different gene selection techniques using 50–200 selected genes.

Method	NBC			SVM			RF			PNN		
Genes selected	50	100	200	50	100	200	50	100	200	50	100	200
Minimum Redundancy–Maximum Relevance (MRMR):	85.22406	89.85625	88.8125	77.6025	76.54	81.51	84.8975	78.90875	76.27	77.02938	85.9375	78.59852
Signal to Noise Ratio (SNR):	76.0675	90.1125	87.5	77.47188	90.88625	77.35758	79.82	92.45	91.67	92.97	88.55	87.10938
Multivariate Error-Weighted Uncorrelated Shrunken Centroid (EWUSC):	85.9375	86.32813	91.47406	77.35758	76.97875	88.025	84.8975	91.1475	85.74344	91.93	89.85625	92.97
Multivariate correlation-based feature selection (CFS):	91.27813	77.3984	90.625	89.85625	75.96875	77.58617	86.32813	77.57801	77.02938	78.97406	87.10938	76.54

**Table 3:** Performance analysis of classifiers in terms of classification accuracies with ABCO for different gene selection techniques using 50–200 selected genes.

Method	NBC			SVM			RF			PNN		
Genes selected	50	100	200	50	100	200	50	100	200	50	100	200
Minimum Redundancy–Maximum Relevance (MRMR):	86.71875	76.81844	88.8125	78.77813	78.6475	87.10938	90.36875	77.21063	85.84047	80.86	84.375	86.32813
Signal to Noise Ratio (SNR):	88.55	85.42	85.74344	89.85625	88.025	90.88625	85.84047	84.22856	80.86	84.08213	91.27813	91.47406
Multivariate Error-Weighted Uncorrelated Shrunken Centroid (EWUSC):	88.8125	81.77	78.59852	85.67875	84.8975	75.75781	75.84375	77.17797	76.96609	76.675	77.73313	76.45563
Multivariate correlation-based feature selection (CFS):	76.21938	76.81	91.1475	85.9375	77.5127	77.53719	88.8125	84.08213	76.84375	77.01672	88.025	89.20625

**Table 4:** Performance analysis of classifiers in terms of classification accuracies with CSO for different gene selection techniques using 50–200 selected genes.

Method	NBC			SVM			RF			PNN		
Genes selected	50	100	200	50	100	200	50	100	200	50	100	200
Minimum Redundancy – Maximum Relevance (MRMR):	78.33727	81.25	91.27813	86.32813	77.11266	77.92906	77.6025	79.82	84.22856	81.77	85.54938	85.84047
Signal to Noise Ratio(SNR):	92.19	90.88625	91.93	77.02938	90.49688	86.71875	85.67875	77.27594	77.39023	86.71875	77.27594	77.39023
Multivariate Error-Weighted Uncorrelated Shrunken Centroid (EWUSC):	83.3495	76.2025	77.47188	77.54535	88.8125	76.05063	79.82	76.37125	83.594	75.75	76.49781	79.69
Multivariate correlation-based feature selection (CFS):	88.55	87.10938	87.10938	89.20625	84.8975	77.97805	77.4882	79.17	89.85625	76.675	81.9	77.39023

**Table 5:** Performance analysis of classifiers in terms of classification accuracies with ICO for different gene selection techniques using 50–200 selected genes.

Method	NBC			SVM			RF			PNN		
Genes selected	50	100	200	50	100	200	50	100	200	50	100	200
Minimum Redundancy–Maximum Relevance (MRMR):	83.3495	85.84047	91.47406	87.10938	76.81	84.22856	77.21063	76.6075	76.23414	86.71875	89.6	85.02813
Signal to Noise Ratio (SNR):	77.36574	75.75	77.32492	77.25961	77.58617	78.71281	75.9375	77.43922	75.875	85.67875	76.90281	76.37125
Multivariate Error-Weighted Uncorrelated Shrunken Centroid (EWUSC):	77.37391	86.71875	88.025	86.52344	90.1125	84.8975	77.39432	76.02531	76.135	75.625	75.75	87.10938
Multivariate correlation-based feature selection (CFS):	82.095	84.08213	80.015	81.77	83.78925	80.015	92.45	90.1125	83.105	76.97875	76.91547	77.47188

**Table 6:** Performance analysis of classifiers in terms of classification accuracies with SSO for different gene selection techniques using 50–200 selected genes.

Method	NBC			SVM			RF			PNN		
Genes selected	50	100	200	50	100	200	50	100	200	50	100	200
Minimum Redundancy–Maximum Relevance (MRMR):	76.81	79.82	85.74344	77.40656	78.33727	76.2025	85.84047	77.37391	76.2025	77.47188	85.74344	77.53719
Signal to Noise Ratio (SNR):	76.92813	77.21063	86.71875	86.32813	87.10938	91.1475	85.67875	77.35758	85.67875	76.54	77.34125	77.47188
Multivariate Error-Weighted Uncorrelated Shrunken Centroid (EWUSC):	83.78925	82.616	86.32813	78.77813	80.73	89.6	81.51	91.1475	94.01125	77.47188	77.39023	78.00254
Multivariate correlation-based feature selection (CFS):	77.63516	77.08	91.27813	91.93	94.53375	94.66438	75.875	85.84047	88.8125	77.78211	94.795	95.705

## Data Availability

The programming codes would be made available to the researchers upon request to the corresponding author.

## References

[1] Alba E., Garcia-Nieto J., Jourdan L., Talbi E. G. Gene selection in cancer classification using PSO/SVM and GA/SVM hybrid algorithms.

[2] Osareh A., Shadgar B. Microarray data analysis for cancer classification.

[3] Haferlach T., Kohlmann A., Schnittger S. (2005). Global approach to the diagnosis of leukemia using gene expression profiling. *Blood*.

[4] Chen Y., Peng C., Sullivan C., Li D., Li S. (2010). Critical molecular pathways in cancer stem cells of chronic myeloid leukemia. *Leukemia*.

[5] Short N. J., Rytting M. E., Cortes J. E. (2018). Acute myeloid leukaemia. *Lancet*.

[6] Haseeb M., Anwar M. A., Choi S. (2018). Molecular interactions between innate and adaptive immune cells in chronic lymphocytic leukemia and their therapeutic implications. *Frontiers in Immunology*.

[7] Hackl H. A. K., Wieser R. (2017). Molecular and genetic alterations associated with therapy resistance and relapse of acute myeloid leukemia. *Journal of Hematology & Oncology*.

[8] Asnafi A. A., Zayeri Z. D., Shahrabi S., Zibara K., Vosughi T. (2019). Chronic myeloid leukemia with complex karyotypes: prognosis and therapeutic approaches. *Journal of Cellular Physiology*.

[9] Lee C.-P., Lin W.-S., Chen Y.-M., Kuo B.-J. (2011). Gene selection and sample classification on microarray data based on adaptive genetic algorithm/*k* -nearest neighbor method. *Expert Systems with Applications*.

[10] Li X., Shu L. (2009). Kernel based nonlinear dimensionality reduction for microarray gene expression data analysis. *Expert Systems with Applications*.

[11] Kumar P. G., Victoire T. A., Renukadevi P., Devaraj D. (2012). Design of fuzzy expert system for microarray data classification using a novel Genetic Swarm Algorithm. *Expert Systems with Applications*.

[12] Sharma S., Imoto S., Miyano A. (2012). Top-r feature selection algorithm for microarray gene expression data. *IEEE/ACM Transactions on Computational Biology and Bioinformatics*.

[13] Aghamaleki F. S., Mollashahi B., Nosrati M., Moradi A., Sheikhpour M., Movafagh A. (2019). Application of an Artificial Neural Network in the Diagnosis of Chronic Lymphocytic Leukemia. *Cureus*.

[14] Binet J., Auquier A., Dighiero G. (1981). A new prognostic classification of chronic lymphocytic leukemia derived from a multivariate survival analysis. *Cancer*.

[15] Afshar S., Abdolrahmani F., Tanha F. V., Seif M. Z., Taheri K. (2011). Recognition and prediction of leukemia with artificial neural network (ANN). *Medical Journal of the Islamic Republic of Iran*.

[16] Wisesty U. N., Warastro R. S., Puspitasari S. Y. (2018). Leukemia and colon tumour detection based on microarray data classification using momentum backpropagation and genetic algorithm as a feature selection method. *Journal of Physics: Conference Series*.

[17] Vogado L. H. S., Veras R. M. S., Araujo F. H. D., Silva R. R. V., Aires K. R. T. (2018). Leukemia diagnosis in blood slides using transfer learning in CNNs and SVM for classification. *Engineering Applications of Artificial Intelligence*.

[18] Kumar M., Rath N. K., Rath S. K. (2016). Analysis of microarray leukemia data using an efficient MapReduce-based K-nearest-neighbor classifier. *Journal of Biomedical Informatics*.

[19] Alrefai N. (2019). Ensemble machine learning for leukemia cancer diagnosis based on microarray datasets. *International Journal of Applied Engineering Research*.

[20] Dwivedi A. K. (2018). Artificial neural network model for effective cancer classification using microarray gene expression data. *Neural Computing and Applications*.

[21] Cho S. B. (2002). Exploring features and classifiers to classify gene expression profiles of acute leukemia. *International Journal of Pattern Recognition and Artificial Intelligence*.

[22] Nasser A. A. E., Shaheen M., Deeb H. E. Enhanced leukemia cancer classifier algorithm.

[23] Huang C. J., Liao W. C. (2004). Application of probabilistic neural networks to the class prediction of leukemia and embryonal tumor of central nervous system. *Neural Processing Letters*.

[24] Nguyen V. D., Rocke D. M. (2002). Tumor classification by partial least squares using microarray gene expression data. *Bioinformatics*.

[25] Golub T. R., Slonim D. K., Tamayo P. (1999). Molecular classification of cancer: class discovery and class prediction by gene expression monitoring. *Science*.

[26] Sewak M. S., Reddy N. P., Duan Z. H. (2009). Gene expression based leukemia sub-classification using committee neural networks. *Bioinformatics and Biology Insights*.

[27] Castillo D., Galvez J. M., Herrera L. J. (2019). Leukemia multiclass assessment and classification from microarray and RNA-seq technologies integration at gene expression level. *PLoS One*.

[28] Dagliyan O., Uney-Yuksektepe F., Kavakli I. H., Turkay M. (2011). Optimization based tumor classification from microarray gene expression data. *PLoS One*.

[29] Pyingkodi M., Thangarajan R. (2017). Meta-analysis in autism gene expression dataset with biclustering methods using random cuckoo search algorithm. *Asian Journal of Research in Social Sciences and Humanities*.

[30] Soto R., Crawford B., Olivares R. (2017). Online control of enumeration strategies via bat algorithm and black hole optimization. *Natural Computing*.

[31] Antonov A. V., Tetko I. V., Mader M. T., Budczies J., Mewes H. W. (2004). Optimization models for cancer classification: extracting gene interaction information from microarray expression data. *Bioinformatics*.

[32] Pyingkodi M., Thangarajan R. (2018). Informative gene selection for cancer classification with microarray data using a metaheuristic framework. *Asian Pacific Journal of Cancer Prevention*.

[33] Lai C.-M., Yeh W.-C., Chang C.-Y. (2016). Gene selection using information gain and improved simplified swarm optimization. *Neurocomputing*.

[34] Tumuluru P., Ravi B. (2018). Chronological grasshopper optimization algorithm-based gene selection and cancer classification. *Journal of Advanced Research in Dynamical and Control Systems*.

[35] Shen Q., Shi W.-M., Kong W. (2008). Hybrid particle swarm optimization and tabu search approach for selecting genes for tumor classification using gene expression data. *Computational Biology and Chemistry*.

[36] Yu H., Gu G., Liu H., Shen J., Zhao J. (2009). A modified ant colony optimization algorithm for tumor marker gene selection. *Genomics, Proteomics & Bioinformatics*.

[37] Nikumbh S., Ghosh S., Jayaraman V. K. Biogeography-based informative gene selection and cancer classification using SVM and random forests.

[38] Tan F., Fu X., Zhang Y., Anu G. (2007). A genetic algorithm-based method for feature subset selection. *Soft Computing*.

[39] Canedo V. B., Maroño N. S., Betanzos A. A. (2012). An ensemble of filters and classifiers for microarray data classification. *Pattern Recognition*.

[40] Salahi M., Jamalian A., Taati A. (2013). Global minimization of multi-funnel functions using particle swarm optimization. *Neural Computing and Applications*.

[41] Emary E., Zawbaa H. M., Hassanien A. E. (2016). Binary grey wolf optimization approaches for feature selection. *Neurocomputing*.

[42] Arora S., Singh H., Sharma M., Sharma S., Anand P. (2019). A new hybrid algorithm based on grey wolf optimization and crow search algorithm for unconstrained function optimization and feature selection. *IEEE Access*.

[43] Ding A., Peng H. Minimum Redundancy Feature Selection from Microarray Gene Expression Data.

[44] Xiong M., Li W., Zhao J., Jin L., Boerwinkle E. (2001). Feature (gene) selection in gene expression-based tumor classification. *Molecular Genetics and Metabolism*.

[45] Yeung K., Bumgarner R. (2005). Correction: multiclass classification of microarray data with repeated measurements: application to cancer. *Genome Biology*.

[46] Wang Y., Tetko I. V., Hall M. A. (2005). Gene selection from microarray data for cancer classification--a machine learning approach. *Computational Biology and Chemistry*.

[47] Odili J. B., Kahar M. N. M. (2016). Solving the traveling salesman’s problem using the African Buffalo optimization. *Computational Intelligence and Neuroscience*.

[48] Karaboga D., Basturk B. (2008). On the performance of artificial bee colony (ABC) algorithm. *Applied Soft Computing*.

[49] Obagbuwa C., Adewumi A. O., Adebiyi A. A. (2014). Stochastic constriction cockroach swarm optimization for multidimensional space function problems. *Mathematical Problems in Engineering*.

[50] Hosseini S., Al Khaled A. (2014). A survey on the imperialist competitive algorithm metaheuristic: implementation in engineering domain and directions for future research. *Applied Soft Computing*.

[51] Cuevas E., Cienfuegos M., Zaldívar D., Pérez-Cisneros M. (2013). A swarm optimization algorithm inspired in the behavior of the social-spider. *Expert Systems with Applications*.

[52] Prabhakar S. K., Rajaguru H., Lee S. W. (2020). A framework for schizophrenia EEG signal classification with nature inspired optimization algorithms. *IEEE Access*.

[53] Prabhakar S. K., Rajaguru H., Lee S.-W. (2019). Metaheuristic-based dimensionality reduction and classification analysis of PPG signals for interpreting cardiovascular disease. *IEEE Access*.

[54] Cuzzocrea A., Francis S. L., Gaber M. M. An information-theoretic approach for setting the optimal number of decision trees in random forests.

[55] Specht A. F. (1990). Probabilistic neural networks. *Neural Networks*.

